# A specialized myodural bridge named occipital-dural muscle in the narrow-ridged finless porpoise (*Neophocaena asiaeorientalis*)

**DOI:** 10.1038/s41598-021-95070-y

**Published:** 2021-07-29

**Authors:** Zhao-Xi Zhang, Jin Gong, Sheng-Bo Yu, Chan Li, Jing-Xian Sun, Shuai-Wen Ding, Guo-Jun Ma, Shi-Zhu Sun, Lin Zhou, Gary D. Hack, Nan Zheng, Hong-Jin Sui

**Affiliations:** 1 Department of Anatomy, College of Basic Medicine, Dalian Medical University, Dalian, China; 2 College of Fisheries and Life Science, Dalian Ocean University, Dalian, China; 3 Department of Engineering Mechanics, Dalian University of Technology, Dalian, China; 4 Department of Advanced Oral Sciences and Therapeutics, University of Maryland School of Dentistry, Baltimore, MD USA; 5 Dalian Hoffen Preservation Technique Institution, Dalian, China

**Keywords:** Anatomy, Musculoskeletal system, Biological physics, Neurophysiology, Animal behaviour, Animal physiology

## Abstract

A dense bridge-like tissue named the myodural bridge (MDB) connecting the suboccipital muscles to the spinal dura mater was originally discovered in humans. However, recent animal studies have revealed that the MDB appears to be an evolutionarily conserved anatomic structure which may have significant physiological functions. Our previous investigations have confirmed the existence of the MDB in finless porpoises. The present authors conducted research to expound on the specificity of the MDB in the porpoise *Neophocana asiaeorientalis (N.asiaeorientalis).* Five carcasses of *N.asiaeorientalis,* with formalin fixation, were used for the present study. Two of the carcasses were used for head and neck CT scanning, three-dimensional reconstructions, and gross dissection of the suboccipital region. Another carcass was used for a P45 plastination study. Also, a carcass was used for a histological analysis of the suboccipital region and also one was used for a Scanning Electron Microscopy study. The results revealed that the MDB of the *N.asiaeorientalis* is actually an independent muscle originating from the caudal border of the occiput, passing through the posterior atlanto-occipital interspace, and then attaches to the cervical spinal dura mater. Thus the so called MDB of the *N.asiaeorientalis* is actually an independent and uniquely specialized muscle. Based on the origin and insertion of this muscle, the present authors name it the ‘Occipital-Dural Muscle’. It appears that the direct pull of this muscle on the cervical spinal dura mater may affect the circulation of the cerebrospinal fluid by altering the volume of the subarachnoid space via a pumping action.

## Introduction

In humans, the suboccipital region is a particularly complex area which contains a connective tissue bridge (MDB) that connects the rectus capitis posterior minor (RCPmi) muscle to the cervical spinal dura mater (SDM). Fascial connections between the suboccipital musculature and the spinal dura mater were noted passing through the atlanto-occipital interspace (in French) by Khan et al.^1^ and later fully described and named the myodural bridge (MDB) by Hack et al.^2^. Subsequent studies revealed that the rectus capitis posterior major (RCPma), the nuchal ligament (NL), and the obliquus capitis inferior (OCI) also participated in the formation of the MDB^3–8^. Moreover, recent studies have evidenced that the MDB exists in numerous mammalian taxa including *Macaca mulatta, Canis familiaris, Felis catus, Oryctolagus cuniculus, Ratus norvegicus, Cavia porcellus, and Indoasian finless porpoise*^9^. In addition, it now has been confirmed that this structure is also present in reptiles (*Crocodylus siamensis*)^10^, and birds (*Columba livia and Gallus domesticus*)^11,12^. The ubiquitous existence of this anatomic structure suggests that the MDB may be a physiologically significant anatomic structure in both humans as well as other species. According to previous morphology studies, the present authors speculated that the MDB is related associated with proprioception^13,14^. Additionally, Zheng et al.^15^ as well as Xu et al.^16^ proposed that the MDB may be a significant factor in modulating the dynamic circulation of cerebrospinal fluid (CSF).

The Narrow-ridged finless porpoise (*N. asiaeorientalis*) is one of the smallest known cetaceans, being marine mammals of the order Cetacea, which includes whales, dolphins, and porpoises^17^. Since 2017, *N.asiaeorientalis* has been placed on the endangered species list by the IUCN Red List of Threatened Species^18^. It has now been shown that finless porpoises represent the most basal clade of porpoises within the family *Phocoenidae*^19–23^. They form a group of mammals that transitioned from the terrestrial to the aquatic environment and now exhibit significant transformations in many of their biological systems. A previous study confirmed the existence of the MDB in *Neophocaena phocaenoides*, however, the Posterior Atlanta-Occipital (PAO) membrane was not observed to be present in that study^24^. In addition, the first three cervical vertebrae in the Finless Porpoise (*N. asiaeorientalis*) are fused together forming a single bony unit^25–27^. Thus, the only remaining passageway for the MDB to reach the SDM is through the atlanto-occipital interspace. More interestingly, the present authors observed a unique and previously unappreciated suboccipital muscle passing through the atlanto-occipital interspace. This previously unidentified muscular structure presumable performs the functional role of a MDB in *N.asiaeorientalis*. Based on these findings, the present study was initiated to further investigate this previously undescribed muscle that passes through the atlanto-occipital interspace of the *N.asiaeorientalis*, confirm the point of attachment of this muscle and observe the detail in ultrastructural level. Also, in order to determine any association between this unique muscle and the MDB observed in humans, as well as to determine the physiological function of this muscle.

## Materials and methods

The analyzed specimens utilized for the present study were narrow-ridged finless porpoises (*N.asiaeorientalis*) that were discovered washed ashore and not living. These specimens were successively collected with permission from the Chinese Authorities for Animal Protection. The present study involving these carcasses was also approved by the Ethics Committee of Dalian Medical University. All the collected bodies were arterially perfused via the aorta with a 10% formalin solution. All the research procedures were carried out in accordance with all relevant guidelines and regulations.

### Methods utilizing CT three-dimensional reconstructions

Two head and neck specimens were scanned utilizing a GE 128-row VCT, and dual phase serial computer tomography (CT) images were obtained, with the slice thickness and pitch being set to 0.6mm. The images were analyzed for modeling and reconstruction with MIMICS software (MIMICS 18.0.0.525, Materialise, Leuven, Belgium).

### Dissection of the postoccipital region

Four specimens were dissected, layer-by-layer, within the posterior occipital region to expose the atlanto-occipital interspace. A dorsal midline incision was made in the neck, and then the skin, subcutaneous fascia, and superficial neck muscles were gradually removed to expose the deep postoccipital musculature. Subsequently, the rectus capitis dorsalis (RCD) muscle was carefully detached from the cranium to reveal the other underlying muscles. The muscles and other anatomic structures within the atlanto-occipital interspace, along with a small segment of the cervical spinal dura mater, were all isolated within tissue blocks using an electric saw. These tissue blocks were then preserved for subsequent histologic evaluation and scanning electron microscopic (SEM) study. Photographic documentation was carried out utilizing a Canon 7D and 450D camera.

### P45 sheet plastination

One specimen of *N.asiaeorientalis* was sliced into sagittal sections for p45 sheet plastination. The obtained P45 sections were semi-transparent with a clear delineation of tissue morphology, including the connective tissues^28^. Anatomical structures in the posterior occipital region and the connections between the postoccipital muscles and the cervical spinal dura mater were observed. This procedure is described as follows^29^:

#### Slicing

The embalmed head and neck specimens were frozen at − 70 ℃ for two weeks and then embedded in polyurethane foam and then again frozen at − 70 ℃ for two days. Afterwards, 3 mm sagittal slices were prepared using a high-speed band saw.

#### Bleaching

The sections were rinsed overnight in cold running water and then immersed in 5% dioxygen for 12 hours.

#### Dehydration

After bleaching, the slices were dehydrated utilizing 100% acetone by the freeze substitution method.

#### Casting and forced impregnation

After dehydration, a casting mold was prepared. The slices were lifted from the acetone bath and placed between two glass plates. The molds were then filled with polyester (Hoffen polyester P45, Dalian Hoffen Bio-Technique Co. Ltd., Dalian, P. R. China.).

The filled molds were then placed upright into a vacuum chamber at room temperature for impregnation. The absolute pressure was slowly decreased from 20 to 0 mm Hg in 5 mm increments, according to the rate of bubble release. The pressure was maintained at 0 mm Hg until bubbling ceased. The impregnation procedure lasted for approximately eight hours.

#### Curing

After the vacuum was released, the air bubbles within the sheets were removed. The top of the mold was clamped with large fold back clamps, and the sheets were then ready for curing. The sheets were cured using a heated water bath and were then placed upright in the water bath at 40℃ for 3 days. After curing, the sheets were removed from the bath and cooled to room temperature in a rack. The slices were then removed from the flat chamber and covered appropriately with adhesive plastic wrap for protection. The sheets were then observed and photographed.

### Histological study

Two tissue blocks were prepared containing the postoccipital musculature, the periosteum of the adjacent cervical vertebrae, the occiput, the adjoining spinal dura mater, and the spinal cord. After being washed in running water overnight, the tissue blocks were then dehydrated with ethanol in increasing grades, passed through xylene, infiltrated, and then embedded in paraffin wax. A rotary microtome was used to cut 10-μm-thick sections. These sections were mounted on glass microscope slides, then rehydrated to prepare for Van Gieson (VG, picric acid and acid fuchsin) staining. The stained sections were analyzed and photographed utilizing a Nikon Eclipse 80i light microscope, with the Nikon NIS image software.

### Scanning electron microscopic study

#### Through layer-by-layer dissections

Two tissue blocks were used for the scanning electron microscope (SEM) study. After washing in running water overnight, the specimens were fixed with 2.5% glutaraldehyde in 0.1 M phosphate buffer at PH 7.3 for approximately 2h. Then, the specimens were repeatedly washed in a buffer solution. They were subsequently dehydrated through a graded alcohol series, vacuum dried with 100% tert-butyl alcohol, and then sputter-coated with platinum using ION SPUTTER JFC-1100 ion sputtering equipment. The specimens were then observed utilizing a scanning electron microscope (model FEI QUATA 200, voltage: 20KV, manufacture: the Netherlands FEI company). The observed fibrous connections were photographed, digitized, and analyzed.

## Results

### CT three-dimensional reconstructions

**Figure 1:**
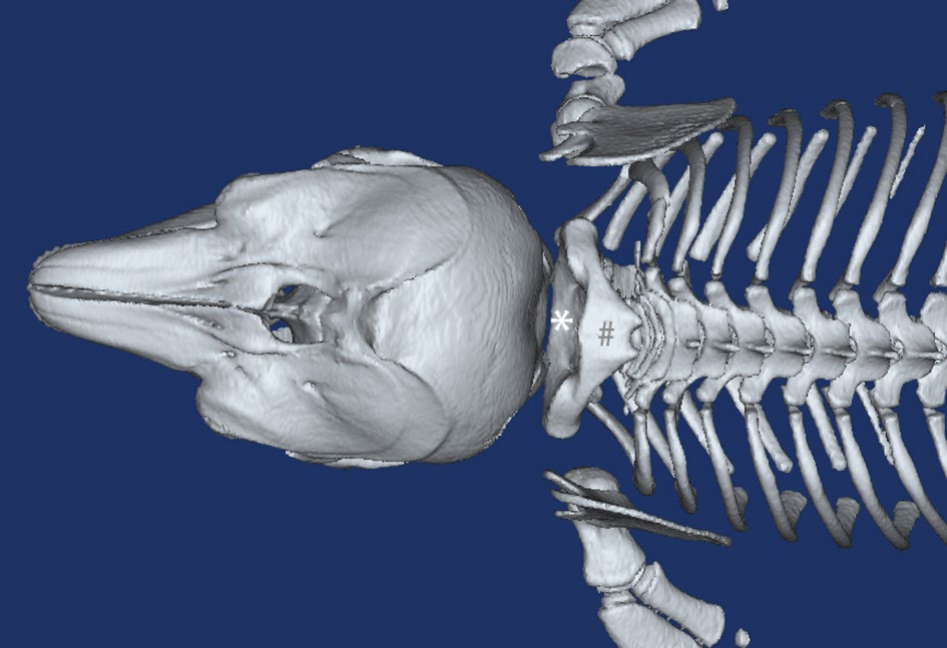
The 3D-reconstruction of bones in suboccipital region of the *N.asiaeorientalis.* Dorsal view of the suboccipital region. occi: occiput. *: the atlanto-occipital interspace. #: the fused spinous processes of the first three vertebrae.

The reconstructed 3D models of the cranium and the cervical vertebrae of *N.asiaeorientalis* evidenced that the atlanto-occipital interspace was broader when compared to that of humans and other terrestrial mammals. The first three cervical vertebrae merged into a single unit. It was observed that both the spinous processes and the transverse processes were fused as well (Fig. 1). As the relationship of these bones was clearly demonstrated by the obtained 3D records, this information was used as a guidance for the gross anatomy aspect of this study.

### Gross anatomy

**Figure 2:**
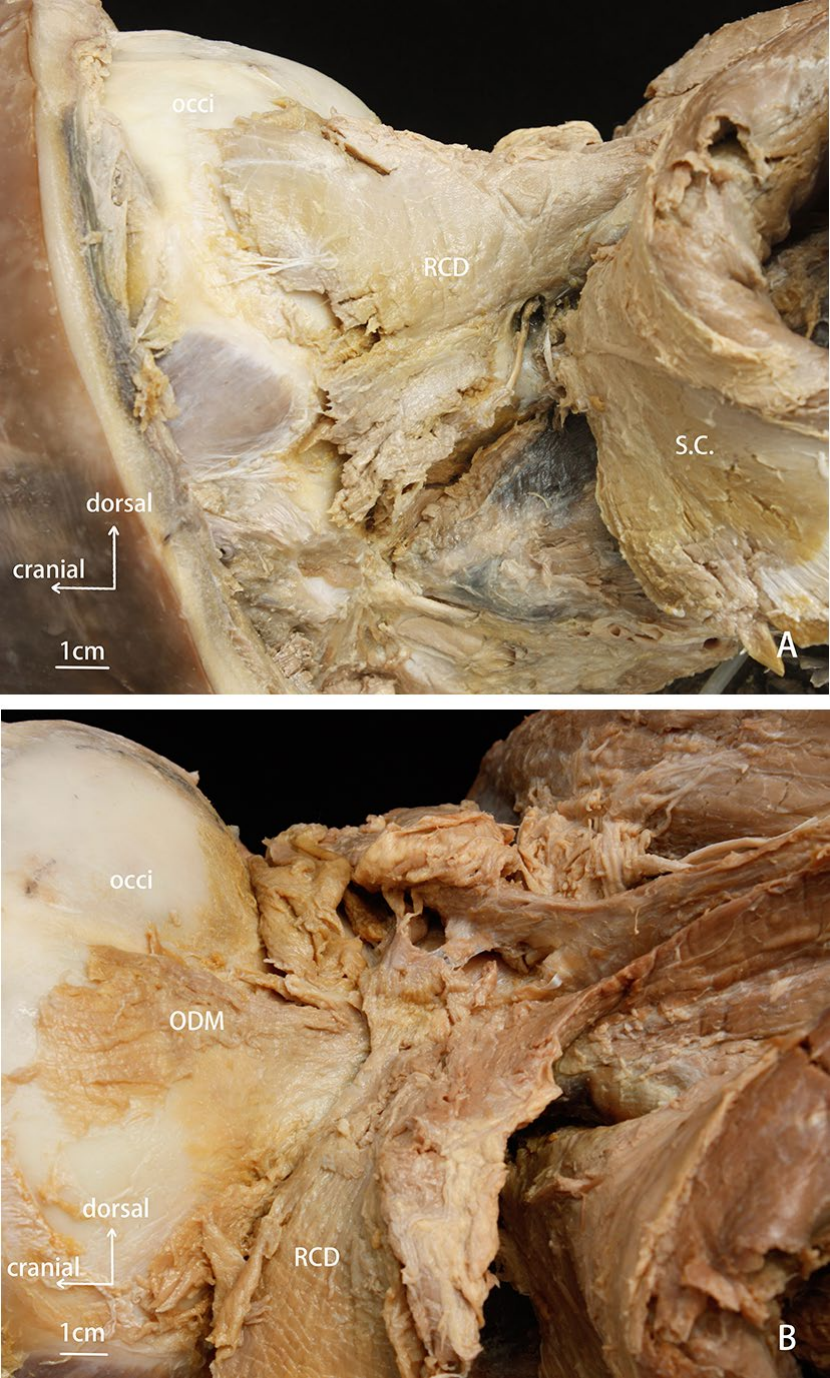
Anatomical dissection of the suboccipital region of the *N.asiaeorientalis*. (**A**) superficial layer of the suboccopital musculature. (**B**) deep layer of the suboccipital musculature. occi: occiput. RCD: rectus capitis dorsalis. S.C.: semispinalis capitis. ODM: occipital-dural muscle.

**Figure 3:**
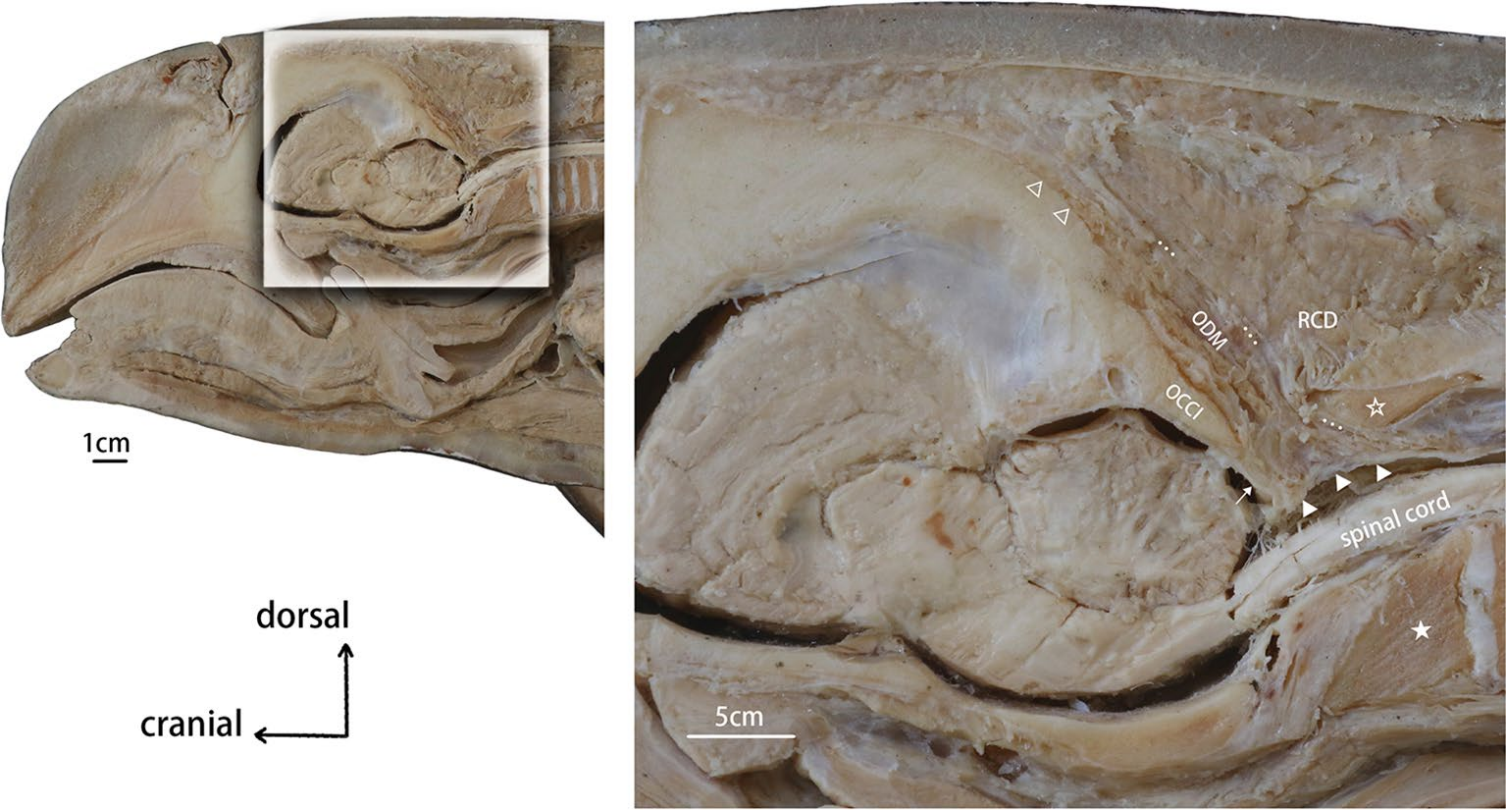
Sagittal section dissection of the suboccipital region of the *N.asiaeorientalis.* OCCI: occiput. RCD: rectus capitis dorsalis. ODM: occipital-dural muscle. ★: the fused vertebral body of the first three vertebrae. ☆: the fused spinous process. △: the origination of the occipital-dural muscle. ▲: termination of the occipital-dural muscle. …: boundary of the occipital-dural muscle. ↑: cerebral dura mater.

With the fusion of the first three vertebrae into a unique single bony unit, the obliquus muscle was observed to not be present in the *N.asiaeorientlis*. The rectus muscle was found in the deep post-occipital region (Fig. 2). The cranial attachment of this rectus muscle was at the occiput, while the caudal attachment was at the transverse process of the fused cervical vertebrae. This muscle was named the rectus capitis dorsalis muscle (RCD). Another muscle was found underneath the RCD, which originated from the occiput, and attached to the spinal dura mater. We named it ‘the occipital-dural muscle’ (Fig. 3). Additionally, the obliquus capitis muscles were found to not be present in the *N.asiaeorientalis.* Moreover, the dorsal atlanto-occipital (DAO) membrane was not found in the *N.aisaeorientalis* during the dissections.

### P45 sheets plastination

**Figure 4:**
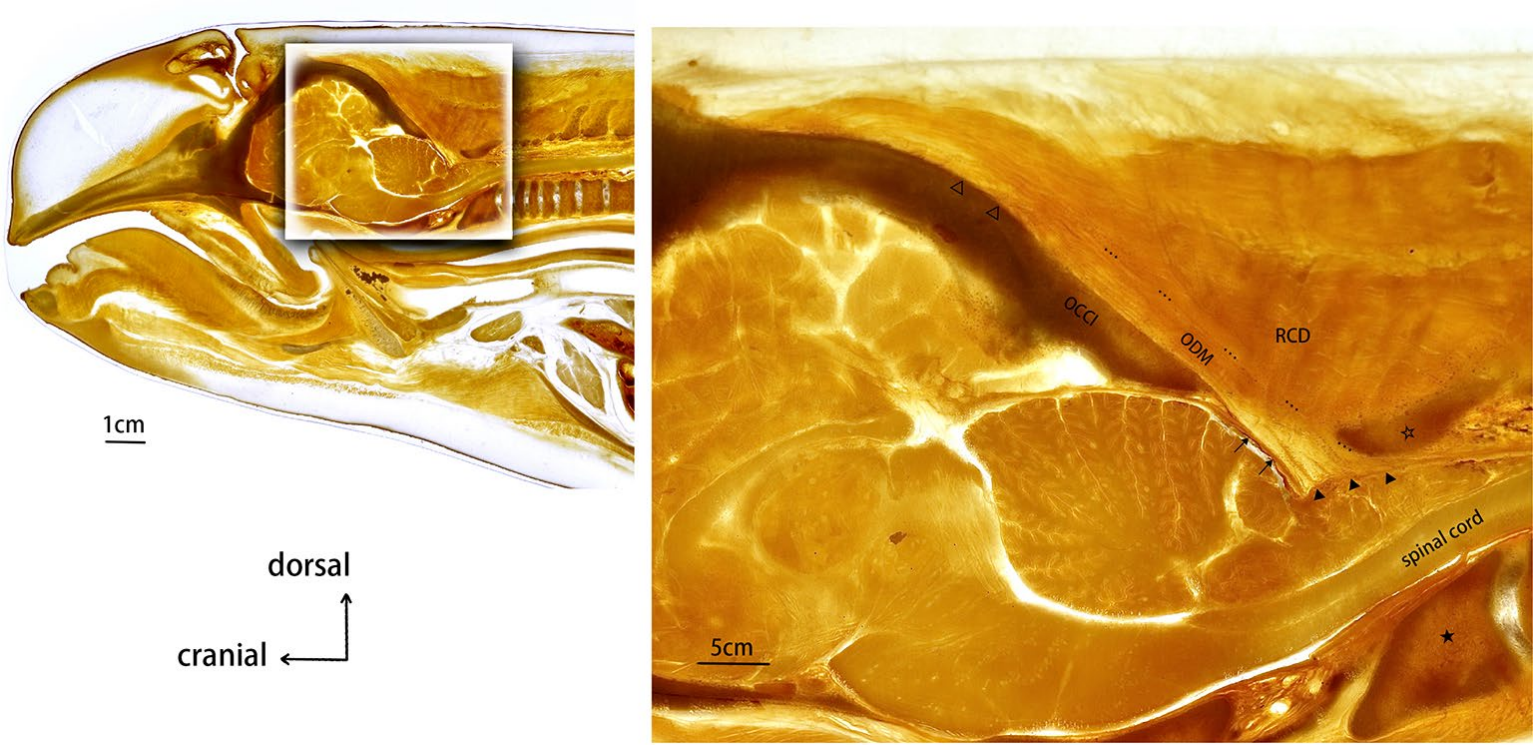
Sagittal section of a P45 plastinated specimen sheet from the suboccipital region of *N.asiaeorientalis.* OCCI: occiput. RCD: rectus capitis dorsalis. ODM: occipital-dural muscle. ★: fused vertebral body of the first three vertebrae. ☆: fused spinous process. △: the origination of the occipital-dural muscle. ▲: termination of the occipital-dural muscle. …: boundary of the occipital-dural muscle. ↑: cerebral dura mater.

Median sagittal sections of the plastinated sheets confirmed that the muscle fibers of the occipital-dural muscle passed through the atlanto-occipital interspace, and attached to the cervical spinal dura mater (Fig. 4). The dorsal atlanto-occipital (DAO) membrane was not found to be present in any of the sheets. In addition, a reverse angle between the cranial and spinal dura mater was observed.

### Histology

**Figure 5:**
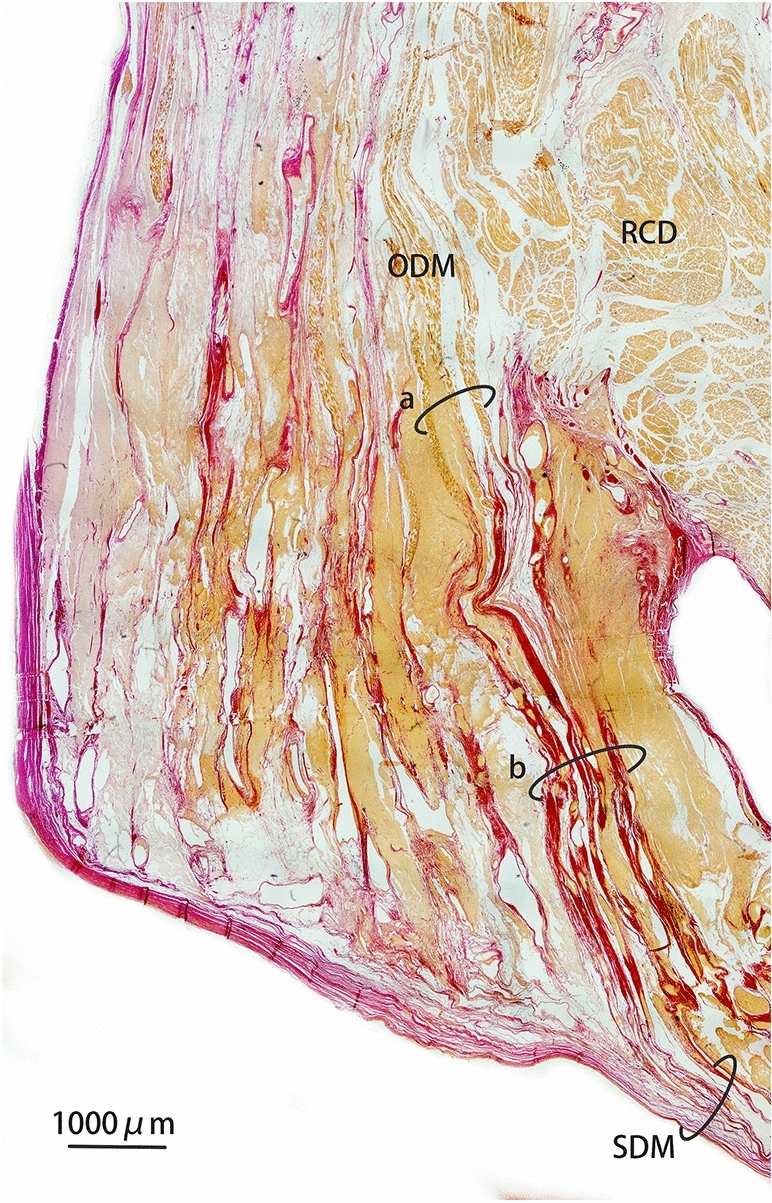
Van Gieson (VG, picric acid and acid fuchsin) stained histological sagittal section of from the suboccipital region of *N.asiaeorientalis*. Images were achieved utilizing a 4 × light microscope. RCD: rectus capitis dorsalis. ODM: occipital-dural muscle. SDM: spinal dura mater. (**a**) fibers of the muscle belly of the occipital-dural muscle. (**b**) fibers of the muscle tendon of the occipital-dural muscle.

Through the histological analysis of VG staining, the relationship between the muscles, bony structures, and the cervical dura mater was clearly identified (Fig. 5). It was found that the proximal attachment of the RCD muscle originated from the occiput while the distal attachment was inserted on to the fused cervical vertebrae. All the muscular fibers of the occipital-dural muscle passed through the atlanto-occipital interspace, and then merged with the spinal cervical dura mater. Additionally, the dorsal atlanto-occipital (DAO) membrane was not observed in any of the histological sections. The sections of VG staining showed detail property of the occipital-dural muscle that the segment passing through the atlanto-occipital interspace were formed by muscular fibers due to their yellow staining, while the segment attaching to the spinal dura mater were stained red, evidencing that these were collagenous fibers of muscle tendon.

### Observation under scanning electron microscope

**Figure 6:**
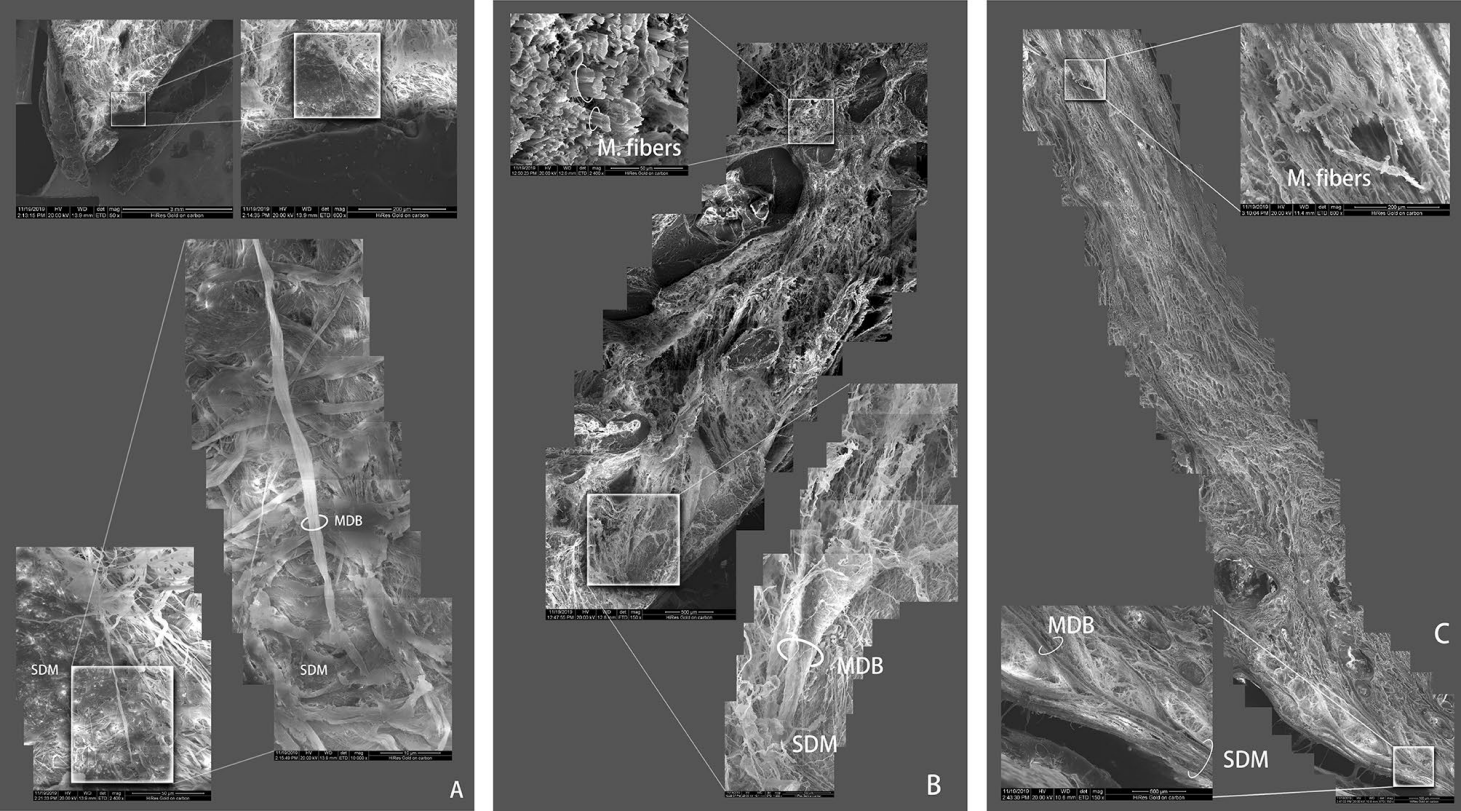
Scanning electron microscopic observation of the occipital-dural muscle and the spinal dura mater of *N.asiaeorientalis* in the atlanto-occipital interspace. (**A**, **B**) connection between the MDB and the SDM. (**C**) sagittal view of a tissue block. MDB: myodural bridge. SDM: spinal dura mater. M. fibers: muscular fibers of the occipital-dural muscle.

The sagittal sections evidenced that the dorsal atlanto-occipital (DAO) membrane was not present in the atlanto-occipital interspace. The SDM was composed of multi-layered fibrous bundles. It was found that the tendonous fibers of the occipital-dural muscle passed through the atlanto-occipital interspace directly, being arranged in parallel bundles, ran caudally, and then ultimately merged with the cervical spinal dura mater. Finally, these tendonous fibers of the occipital-dural muscle formed the superficial layer of the SDM. Also, some tendonous fibers interweave with the fibers composing the SDM (Fig. 6).

## Discussion

The myodural bridge was initially described as a dense fibrous connection between the rectus capitis posterior minor muscle and the SDM by Hack et al.^2^. Subsequent studies built upon this original study. The present study is the first to identify a species possessing an MDB structure that actually exists as a unique muscle that inserts into the SDM via its muscular tendon. Considering the physiological significance of the MDB in humans, Sui et al.^30^ and Zheng et al.^15^ proposed that the suboccipital muscles connecting to the upper cervical spinal dura mater via the MDB provides a power source for the circulation of cerebrospinal fluid (CSF). Moreover, Xu et al.^16^ postulated that head movements could be significant contributor to CSF dynamics at the craniocervical junction, in addition to other factors such as heartbeat^31,32^ and respiration^33–35^.

Finless porpoises have a wide geographical range existing in shallow, coastal waters^26,36^. The narrow-ridged finless porpoise (*N.asiaeorientalis*) has been designated as a new species by the International Union for Conservation of Nature (IUCN). This porpoise species is endemic to the western Yangtze river in China, the East China Sea, the Yellow Sea, and around Japan. Narrow-ridged finless porpoises (*N.asiaeorientalis*), having no dorsal fin and a flexible neck, which is narrower than that of other porpoise species. These porpoises have tubercles on its their backs from mid-back to their tail, with a dorsal ridge ranging from 0.2 to 1.2 cm wide^37^. Our team previously confirmed that the MDB exists in finless porpoises (*N.phocaenoids*), and in the sperm whale^24,38^. Like most marine mammals, *N.asiaeorientalis* must hold their breath during dives while foraging in their aquatic habitats. During these dives, they can experience extensive apnea, similar to that of their related species, harbor porpoises^39,40^. To tolerate the extreme conditions of deep diving, a response evolved, which consists of bradycardia and peripheral vasoconstriction. As a result of this, transient cessation of respiration occurs, cardiac output and organ perfusion are diminished^40–42^. However, these processes do not occur in terrestrial mammals. Thus, compared to their terrestrial counterparts, marine mammals routinely confront physiologic challenges that stem from their response to diving. Although blood pressure and respiration are considered to be crucial for maintaining adequate circulation of the CSF, marine mammals experience a decrease in their heart rate and respirations during dives.

The present study found the MDB in *N.asiaeorientalis* to be an independent muscle called occipital-dural muscle that originates from the occiput, passes through the atlanto-occipital interspace, and terminates on the spinal dura mater directly. Previously, an analogous structure was found in *N.phocaenoids,* without the existence of a PAO membrane serving as an intermediate tissue^24^. Neither could we find any other termination of this independent muscle except the SDM in *N.asiaeorientalis*. While previous investigation did not give a clear description about this connection between this muscle and the cervical spinal dura mater.

As previous research has reported, the MDB was found to exist in the vast majority of studied mammals^9^, with the MDB now considered an evolutionally conserved structure. The analogous MDB of *N.asiaeorientalis* is a unique structure among the studied animals we have examed.

Previous studies in humans showed that in atlanto-occipital interspace, the connection between the RCPmi and the SDM was indirect because of the existence of PAO membrane under the scanning electron microscope observations^43^. Also, only part of fibers originated from RCPmi formed MDB. The termination of RCPmi is still on the atlas. While in *N.asiaeorientalis*, as the first three cervical vertebrae fused together and formed a single bony unit, the atlanto-occipital interspace became the only passageway for the suboccipital muscle to reach the SDM. This muscle has no other termination. Due to the absence of a PAO membrane, the occipital-dural muscle in the *N.asiaeorientalis* passes unimpeded through the atlanto-occipital interspace, and attaches to the SDM directly. In addition, the present scanning electron microscope results showed that the fiber bundles of the occipital-dural muscle passed through the atlanto-occipital interspace, ultimately merged with the cervical spinal dura mater, formed as a part of the SDM, obviously revealed that the occipital-dural muscle and the SDM could not be separated at the ultrastructural level. Thus, we speculated that the traction force of the occipital-dural muscle could directly act on the SDM. With the contraction and the relaxation of the occipital-dural muscle, creating alternating pumping forces on the SDM appears to be a significant functional role for the occipital-dural muscle in *N.asiaeorientalis*. During the finless porpoise dive time, both the lower heart rate and the suspended respiration decreases this important power source for maintaining the CSF movement. Therefore, providing powerful tractional forces to the SDM during movements between the head and neck might be the only function for this specialized muscle.

In humans, the vertebral venous plexus is located between the periosteum of the vertebra and the SDM. The vertebral venous system can buffer the changes in intracranial pressure caused by respiration and heartbeats, which is an important channel for regulating intracranial pressure and blood circulation^44,45^. Both intracranial pressure and intracranial blood circulation are important influencing factors of CSF circulation. A large number of venous plexus filling in the tissue of the MDB was found in *N. phocaenoides*^24^, while present study did not notice, which might suggest empty and reflow of the venous blood. The vascular plexus among the fibers of the MDB were presumed to be the intervertebral venous plexus of the finless porpoise based on its location. When swimming in deep water, its heart rate slowed and breathing stopped. In the meantime, the contraction of the occipital-dural muscle might drive the venous blood of intervertebral venous plexus among the tendons to empty and reflow. Therefore, for the finless porpoise, the occipital-dural muscle appears to be an important regulatory structure that might be able to buffer the changes in intracranial pressure caused by respiration and heartbeats, which affects the CSF circulation.

Therefore, this unique muscle in *N.asiaeorientalis* may play an indispensable role providing an additional mechanism for the dynamic circulation of the CSF. Moreover, this proposed mechanism would be continuously sustained during dives due to the finless porpoises’ continuous body motion. However, for marine mammals, current investigations of the MDB are only initiated on finless porpoise and sperm whale. The limited number and narrow comparison lead to the uncertainty when discussing the association between their anatomical modifications and dive response.

**Figure 7:**
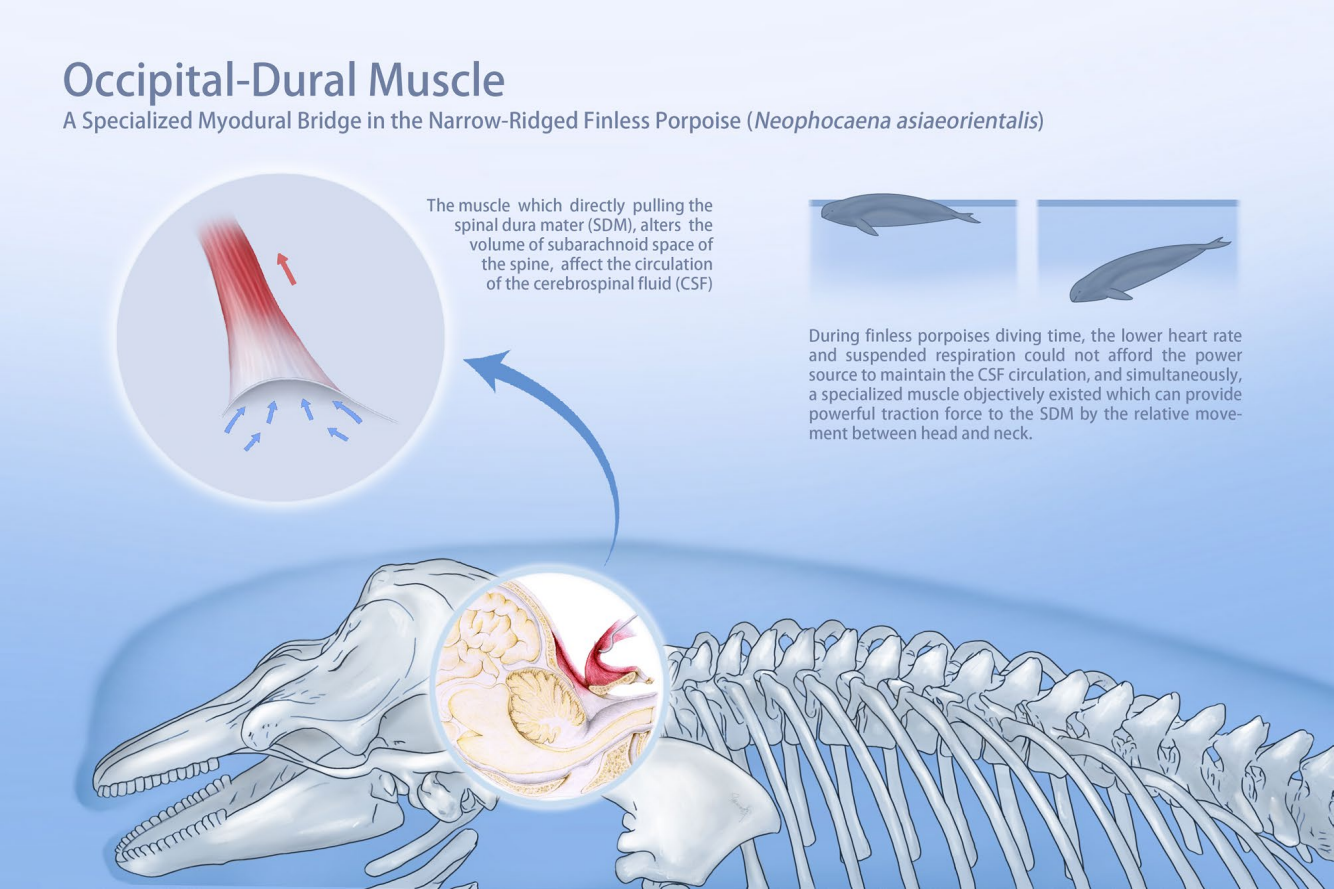
Image demonstrates the occipital-dural muscle pulling on the spinal dura mater (SDM), which alters the volume of fluid within the subarachnoid space, thus affecting the circulation of the cerebrospinal fluid (CSF).

In summary, the evidence supports that the MDB observed in *N.asiaeorientalis* is an isolated functioning muscle that we named the occipital-dural muscle. Unlike the MDB in humans, this muscle directly connects to the SDM in *N.asiaeorientalis*, and may transmit strong tractional forces to the SDM, by muscular contraction and relaxation. Furthermore, this mechanism is associated with the movement between the occiput and the fused first three cervical vertebrae (Fig. 7). This mechanism appears to be more powerful and significant than that in humans and most of other species that have been previously investigated. The present findings enhance the credibility and further validates the significance of the proposed physiological function of the MDB or its analogous muscular structure described in this paper for the first time.
